# Epigenetic regulation of cancer biology and anti-tumor immunity by EZH2

**DOI:** 10.18632/oncotarget.12928

**Published:** 2016-10-26

**Authors:** Anthos Christofides, Theodoros Karantanos, Kankana Bardhan, Vassiliki A. Boussiotis

**Affiliations:** ^1^ Division of Hematology-Oncology, Beth Israel Deaconess Medical Center, Boston, MA, USA; ^2^ Department of Medicine Beth Israel Deaconess Medical Center, Harvard Medical School, Boston, MA, USA; ^3^ General Internal Medicine Section, Boston Medical Center, Boston University School of Medicine, Boston, MA, USA

**Keywords:** cancer biology, epigenetics, EZH2, cancer immunology

## Abstract

Polycomb group proteins regulate chromatin structure and have an important regulatory role on gene expression in various cell types. Two polycomb group complexes (Polycomb repressive complex 1 (PRC1) and 2 (PRC2)) have been identified in mammalian cells. Both PRC1 and PRC2 compact chromatin, and also catalyze histone modifications. PRC1 mediates monoubiquitination of histone H2A, whereas PRC2 catalyzes methylation of histone H3 on lysine 27. These alterations of histones can lead to altered gene expression patterns by regulating chromatin structure. Numerous studies have highlighted the role of the PRC2 catalytic component enhancer of zeste homolog 2 (EZH2) in neoplastic development and progression, and EZH2 mutations have been identified in various malignancies. Through modulating the expression of critical genes, EZH2 is actively involved in fundamental cellular processes such as cell cycle progression, cell proliferation, differentiation and apoptosis. In addition to cancer cells, EZH2 also has a decisive role in the differentiation and function of T effector and T regulatory cells. In this review we summarize the recent progress regarding the role of EZH2 in human malignancies, highlight the molecular mechanisms by which EZH2 aberrations promote the pathogenesis of cancer, and discuss the anti-tumor effects of EZH2 targeting via activating direct anti-cancer mechanisms and anti-tumor immunity.

## INTRODUCTION

The term *Polycomb* (*Pc*) initially referred to a *Drosophila* mutant that displayed improper body segmentation. Based on this observation it was suggested that Polycomb encodes a negative regulator of the homeotic genes that are required for segmentation [1]. Now, it is known that the Polycomb group (PcG) defines a set of genes characterized by mutations that result in similar phenotypes to those of Polycomb but has extended its functions from Drosophila to mammalian cells [2]. The crucial role of PcG proteins during development is highlighted by early embryonic lethality in mice after the deletion of genes encoding some of these proteins (*Eed, Ezh2* (also known as *Enx-1*), *Suz12* and *Ring1B* (*Rnf2*)).

**Figure 1:**
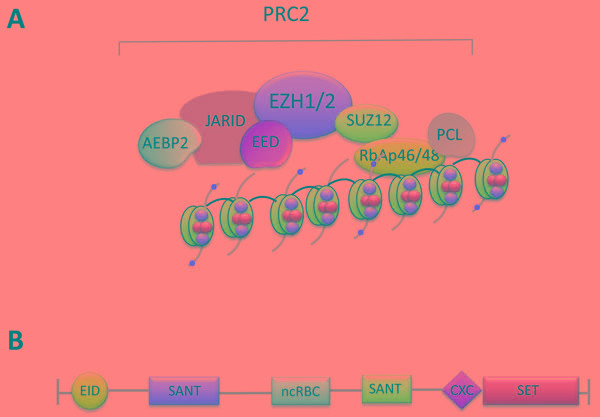
**A.** The polycomb complex PRC2. PRC2 contains EZH2 or EZH1 and four key components, EED, EZH1/2, Suz12 and RbAp46/48, which are required for its enzymatic activity. AEBP2, PCL and JARID2 are also components of the PRC2 complex but their precise role is currently unclear. **B.** Characterized domains of EZH2. EID, EED interaction domain; SANT, SWI3, ADA2, N-CoR and TFIIIB DNA-binding domain; ncRBD, non-coding-RNA-binding domain; CXC, cysteine-rich domain; SET, Su(var)3-9, enhancer of zeste, trithorax domain.

PcG proteins are found in several families of multiprotein complexes, including the Polycomb repressive complexes, which form two closely related complexes, PRC1 and PRC2. PRCs mediate gene silencing mainly by regulating chromatin structure through posttranslational modification (PTM) of histones. The PRC2 complex is responsible for trimethylation of Lys 27 of histone H3 (H3K27me3) through its enzymatic subunits EZH1 and EZH2, which are core components of PRC2. H3K27me3 plays an important role in epigenetic gene silencing, particularly in homeobox (Hox) gene loci. The methyltransferase activity of EZH2 is provided by the signature SET domain, located at the COOH-terminus. Importantly, EZH2 lacks enzymatic function and must partner with at least three noncatalytic proteins, including EED, SUZ12 and RbAp48/46, to form the PRC2 complex, which has potent histone methyltransferase activity [3] (Figure 1). Subsequently, it was determined that AEBP2, PCL and JARID2 are also components of the PRC2 complex. Although these additional components might be necessary for optimal PRC2 activity, their precise role has not been fully elucidated. EZH1 also forms a PRC2 complex. EZH2 is present only in dividing cells, whereas EZH1 is found both in dividing and non-dividing cells. The PRC2 complexes, which contain EZH1, have low methyltransferase activity compared to PRC2 complexes, which contain EZH2. The PRC1 complex is responsible for monoubiquitylation of Lys 119 of histone H2A (H2AK119ub) via the ubiquitin ligases RING1A and RING1B. PRC1 occupies some of the PRC2 sites [4] and may function downstream of PRC2 [2]. In contrast to the limited knowledge about the role and functions PRC1 in mammalian cells, PRC2 has been extensively studied and its key role in cell differentiation and fate determination is well established. The PRC2 complex has evolved not only as a key regulator of cell differentiation and plasticity during evolution but also as a molecular mediator of cancer development and a novel target of cancer treatment.

The sequencing of the cancer genome has revealed that genes encoding chromatin regulators are frequently mutated in solid tumors and hematologic malignancies highlighting the role of these molecules in the development and progression of human neoplasms [5-7]. Alterations of histone methylation and acetylation mediated by chromatin modulators can lead to down-regulation of tumor suppressor genes and overexpression of oncogenes implicated in the development and progression of malignancies [8]. Moreover, chromatin modulators are involved in oncogenic signaling independently of their role in epigenetic regulation and have been directly associated with activation of intracellular pathways leading to cell proliferation, apoptosis inhibition and cancer progression. Several recent studies have highlighted the role of EZH2 in neoplastic development and progression while this molecule has been associated with poor prognosis in a variety of human malignancies.

EZH2, the catalytic subunits of the PRC2 catalyzes the methylation of histone H3 lysine 27 to form H3K27me3 by its C-terminal SET domain [9], promoting transcriptional silencing through development of repressive marks on the promoters of target genes [2]. Through modulating the expression of critical genes, EZH2 is actively involved in fundamental cellular processes such as cell cycle progression, cell proliferation, cell differentiation and apoptosis. EZH2 mutations have been found in various malignancies, while EZH2 expression levels are associated with the prognosis of human neoplasms suggesting the potential involvement of EZH2 in the development and progression of cancer [10]. The interest in better understanding of the role of EZH2 in cancer has been enhanced by the development of novel agents that inhibit EZH2 enzymatic activity, which are currently under evaluation in early phase clinical trials showing promising results.

## EVIDENCE CORRELATING EZH2 FUNCTION WITH CANCER

### Solid tumors

Increased EZH2 expression was initially demonstrated in prostate and breast cancer based on microarray studies which supported the hypothesis that the upregulation of this protein is associated with advanced stage and poor prognosis [11, 12]. Subsequent studies showed that EZH2 is upregulated in various solid malignancies including renal cell carcinoma [13], lung [14, 15], hepatocellular [16], gastric [17], and pancreatic cancer [18]. Recent studies have highlighted the prognostic significance of EZH2 expression in a variety of solid cancers. Particularly, Wang et al in a meta-analysis study showed that EZH2 overexpression correlates with poor prognosis in patients with non-small cell lung cancer [19] while Bachmann et al in a prospective analysis of 700 cancer patients demonstrated that EZH2 is a marker of increased aggressiveness in patients with melanoma and endometrial cancer consistently with the previous findings in patients with breast and prostate cancer [19, 20]. In the later study the authors found that EZH2 expression was strongly associated with loss of the cell cycle suppressor protein p16 in melanomas and endometrial carcinomas and increased expression in cyclin D1 in melanomas [20] connecting their findings with uncontrolled cell cycle in cancer cells overexpressing EZH2. EZH2 expression in colorectal cancer was reported to positively associate with TNM stage and lymph node metastasis [21] while another study evaluating the role of EZH2 expression in gastrointestinal malignancies, found that its overexpression is correlated with poor prognosis in esophageal but not colorectal and gastric cancer [22]. Moreover, EZH2 has been strongly associated with advanced stage and poor prognosis in squamous cell malignancies including cervical cancer [23], esophageal squamous cell carcinoma [24] and head and neck cancer [25]. Particularly, EZH2 promotes the epithelial to mesenchymal transformation (EMT) in head and neck carcinomas and decreases the sensitivity to cisplatin-based chemotherapy [25].

## HEMATOLOGIC MALIGNANCIES

### Leukemia

As opposed to solid tumors where EZH2 overexpression is clearly associated with disease progression and poor prognosis, data are not so clear in hematologic malignancies. Both overexpression [26] and loss-of-function mutations [27, 28] have been detected in myelodysplastic syndrome (MDS) and acute myeloid leukemia (AML) suggesting that EZH2 can function as tumor suppression and as an oncogene in myeloid malignancies. Tanaka et al proposed that EZH2 reinforces the differentiation blockage maintaining the self-renewal capacity of leukemic cells in AML [29]. Recent data suggest that EZH2 loss is more strongly associated with myelodysplastic syndromes, particularly, chronic myelomonocytic leukemia. It has been shown that EZH2 loss promotes the development of MDS in a RUNX1S21fs mutant model but inhibits the transformation to AML [30]. The authors suggested that despite the compromised proliferative capacity in the setting of EZH2 loss, induction of cytokine production in the tumor microenvironment might lead to expansion of the dysplastic myeloid clones while upregulation of EZH2 can promote progression to AML [30]. Similarly, Muto et al supported that deletion of EZH2 was sufficient to induce MDS/Myeloproliferative neoplasm (MDS/MPN) in transgenic mice [31], whereas Soverini et al reported loss-of-function mutations of EZH2 gene in chronic myeloid leukemia [32] further supporting the hypothesis that EZH2 inactivation may be implicated in chronic myeloid dysplasia and hyperplasia. On the contrary, EZH2 is frequently overexpressed in AML with complex phenotype [33], whereas EZH2 deletion decreases proliferation and promotes apoptosis in MLL-AF9 transformed cells, and delays the progression of AML in transgenic mice [29]. Similarly, increased EZH2 expression is associated with extramedullary infiltration in AML and activation of ERK/c-Myc/MMP-2 and E-cadherin signaling [34].

### Non-Hodgkin Lymphomas

EZH2 seems to have an active role in lymphoid malignancies since EZH2 gain-of-function mutations have been identified in germinal center B cell diffuse large-cell B cell lymphomas (DLBCL) and follicular lymphomas [35, 36]. It has been determined that EZH2 methyltransferase activity is mandatory for the coordinated regulation of GC B cell differentiation, proliferation, and response to genotoxic damage imposed by activation-induced cytidine deaminase (AID). Conversely, enforced BLIMP1 repression, coupled to protection against AID mutagenesis, acceleration of S-phase entry, and enhanced BCL6 function, may represent the mechanism through which constitutively active EZH2 contributes to lymphomagenesis [37]. These results provide a basis for using EZH2 inhibitors [38-40] in combination with inducers of terminal B cell differentiation, such as IL-21 [41, 42] and genotoxic agents, for the treatment of GC-derived DLBCL and follicular lymphoma.

Together these reports support the conclusion that EZH2 is dysregulated in a large spectrum of human malignancies and this event has prognostic significance. The mechanisms of EZH2 aberration and the molecular pathways involved in the regulation of EZH2 abundance and activity in human neoplasms together with the development of novel therapeutic opportunities to target EZH2 are under intense investigation.

## DEREGULATION OF EZH2 FUNCTION IN CANCER

EZH2 expression and activity in cancer cells is altered at genetic, transcriptional, post-transcriptional and post-translational levels. The complexity of EZH2 regulation by multiple molecular pathways, most likely contributes to the variable implications of EZH2 aberrations in various types of cancer.

### EZH2 mutations

Several recent studies have shown that heterozygous point mutations affecting tyrosine 641 (Y641) within the C-terminal catalytic SET domain of EZH2 have been identified in 22% of germinal center B cell diffuse large-cell B cell lymphomas (DLBCL) and in 7-12% of follicular lymphomas [35, 36]. This mutation mediates gain-of-function of EZH2 enzymatic activity leading to increased levels of trimethylated H3K27 (H3K27me3) [43] and resulting in down-regulation of Polycomb target genes such as *TCF4, FOXP1, TCL1A, BIK*, and *RASSF6P* in follicular lymphomas [44]. Other studies have identified additional EZH2 gain-of-function point mutations at the alanine 677 and 687 residues in non-Hodgkin's lymphomas, which are associated with increased H3K27 trimethylation [45, 46]. Although the outcome of these gain-of-function mutations has documented a role of EZH2 as an oncogenic mediator, other findings suggest that EZH2 might function as a tumor suppressor because EZH2 loss-of-function is associated with development of malignancySpecifically, EZH2 inactivating deletion, frameshift, nonsense and missense mutations have been identified in myelodysplastic syndromes (MDS), myeloproliferative neo­plasms (MPN) and MDS-MPN overlap disorders [27, 28]. Consistently with these findings in patients, indicating a tumor-suppressive role of EZH2 in MDS, mice lacking *Ezh2* have enhanced initiation and progression of *Runx1*-mutant MDS [30]. Loss-of-function mutations and deletions of *EZH2* have also been identified in T cell acute lymphoblastic leukemia (T-ALL) [47]Since EZH2 loss-of-function can drive oncogenesis of certain cancers, caution is required during therapeutic application of EZH2 inhibitors.

### Transcriptional regulation

There is strong evidence that oncogenic signaling in a variety of human neoplasms leads to alteration of EZH2 transcriptional regulation promoting cancer cell proliferation and disease progression. The fusion protein EWS-FLI1 in Ewing's sarcoma cells promotes the expression of EZH2 and has been associated with endothelial/neuroectodermal differentiation in this type of tumor [48]. Similarly, RAS gain-of-function mutations have been associated with elevated EZH2 expression in various malignancies. In pancreatic cancer, RAS mutation promotes the expression of EZH2 through activation of the MEK-ERK-ELK1 signaling [49]. Activation of MEK-ERK signaling pathway has also been associated with EZH2 upregulation in triple negative and ERBB2 positive breast cancer [50]. It is believed that the phosphorylated ELK1 downstream of ERK binds to the *EZH2* gene promoter activating its transcription [10, 51]. It has been determined that different amino acid substitutions in oncogenic KRAS differently modulate EZH2 expression in lung adenocarcinoma showing that KRAS^G12C^ is associated with higher EZH2 expression [52]. This study also determined that different KRAS mutations can promote the expression of EZH2 through PI3K-AKT or MEK-ERK signaling in non-small cell lung cancer, a finding that allows for therapeutic combinations of pathway targeting agents, such as AKT and EZH2 inhibitors or MEK and EZH2 inhibitors, to achieve maximum therapeutic benefit in cancers which display joint aberration of these targets [52].

The Rb-E2F signaling has also been found to activate the expression of EZH2 through direct binding of E2F on the *EZH2* promoter upon Rb/RB1 phosphorylation in bladder and small cell lung cancer [53, 54]. Hypoxia, which is a known driver of solid tumor progression, promotes EZH2 expression through HIF-1α binding on the *EZH2* promoter, inducing disease aggressiveness and progression [55]. Dong et al recently demonstrated that breast cancers overexpressing both *HIF-1*α and *EZH2* have poorer overall survival compared to cancers overexpressing one of these genes [56]. Importantly, in prostate cancer EZH2 is decreased by androgens, while androgen deprivation increases the expression of EZH2, which downregulates E-cadherin thereby promoting cell migration and cancer progression [57]. These findings provide better understanding of EZH2 regulation at the transcriptional level and justify the use of EZH2 inhibitors in combinational approaches for targeted cancer therapy.

### Post-transcriptional regulation

The expression of EZH2 is also regulated by post-transcriptional mechanisms through micro-RNAs. Particularly, micro-RNAs (miRs) can bind to the EZH2 RNA transcript and modulate its stability, integrity and translation, directly affecting the levels of EZH2 protein. Multiple miRs such as miR-25, -26A, -101, -138 and -214 interact with the EZH2 3’UTR and promote its degradation [51, 58]. Down-regulation of these miRs can lead to increased expression of EZH2 and may have implications in disease progression. Zhu et al recently found that miR-138, which is down-regulated in osteosarcoma, directly targets EZH2 transcript and can increase the sensitivity of osteosarcoma cells to cisplatin [59]. Other studies have identified a double-negative feedback loop between EZH2 and miR-26a in hepatocellular cancer, since miR-26a, which directly targets EZH2 at a post-transcriptional level, is also abrogated by EZH2 overexpression [60]. Similarly, down-regulation of miR-101 in glioblastoma cells promotes their proliferation and invasion along with angiogenesis by increasing the EZH2-mediated overexpression of cytoplasmic polyadenylation element-binding protein 1 (CPEB1) [61, 62]. These findings were further confirmed in astrocytic tumors in which it was determined that a network of interactions between EZH2 and miR-26a, -27a and -498 is differentially expressed in low and high grade tumors. Detection of this EZH2 network may serve as a promising biomarker for tumor progression into higher grade [63].

Post-transcriptional regulation of EZH2 is also mediated by additional mechanisms. AKT1-mediated phosphorylation of EZH2 allows its interaction with androgen receptor promoting the expression of androgen receptor-target genes in the absence of androgens further supporting the involvement of EZH2 in the development and progression of castrate resistant prostate cancer [64]. Similarly, AKT1 phosphorylates EZH2 and induces the expression of STAT3 in glioblastoma with stem-like cells promoting tumorigenicity [65]. Cyclin-dependent kinases 1 and 2 (CDK1/2) phosphorylate EZH2 at T350 increasing its recruitment at chromatin and positively affecting its stability and activity [66]. Phosphorylation of EZH2 by CKD1/2 at T492 disrupts the PRC2 assemblies and decreases EZH2 activity [67]. Moreover, CKD1 phosphorylation of EZH2 at T350 and T492 promotes EZH2 ubiquitinylation and degradation suggesting decreased methyltransferase activity [67]. While it is clear that CDK mediated EZH2 phosphorylation is critical for the maintenance of H3K27me3 marks through the cell cycle, the role of this interaction in the fate and function of cancer cells warrants further investigation. Hussain et al demonstrated that exposure to tobacco smoke condensate leads to EZH2 binding on the promoter of the Wnt-antagonist, Dickorpf-1, inhibiting its transcription and activating oncogenic Wnt signaling in lung cancer cells [68]. These findings suggest a possible effect of an exogenous carcinogen through post-translational modification of EZH2. Taken together these studies strongly suggest that regulation of EZH2 at the post-translational level might be of particular importance with regards to its activity and implication in cancer development and growth.

## CANCER CELL-INTRINSIC MECHANISMS OF EZH2-MEDIATED ONCOGENESIS

**Figure 2:**
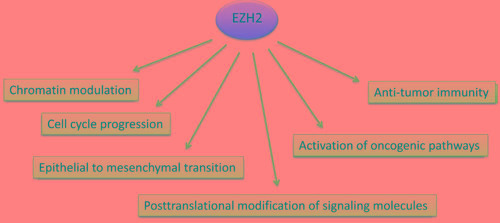
Mechanisms of EZH2-mediated implications in cancer EZH2 expression and activity in cancer cells is altered at the genetic, epigenetic and post-translational levels. Such changes activate oncogenic genes and pathways thereby promoting tumorigenesis, and alter cell cycle progression and metastasis of cancer cells. EZH2-mediated alterations in cancer cells affect the recruitment of anti-tumor T cells in the tumor microenrvironment in response to tumor-immunotherapy.

Although the implication of EZH2 in the development and progression of malignancies has been extensively studied and well documented, the mechanism(s) involved remain uncertain. It is believed that EZH2 affects the pathogenesis of cancer by multiple different mechanisms as it is involved in a various cell intrinsic molecular pathways (Figure 2). Particularly, as part of PRC2, EZH2 can affect the expression of numerous target genes that are critical for the survival, proliferation and aggressiveness of cancer cells, via epigenetic mechanisms. In parallel, there is accumulating evidence that EZH2 is also involved in other oncogenic pathways that are not implicated in chromatin modulation. Moreover, EZH2 activity might have significant impact on immune-mediated tumor distraction in response to immunotherapy with checkpoint inhibitors.

### Chromatin modulation

Since EZH2 suppresses the differentiation of normal embryonic stem cells by decreasing the expression of lineage specifying factors [69], it is not surprising that it is highly expressed in cancer stem cell (CSC) populations [55, 70] maintaining their survival and inhibiting their differentiation [71, 72]. It is believed that EZH2 as part of the PRC2 suppresses cell specific transcriptional programs that mediate differentiation or apoptosis such as GSK-3β and p53 in cervical cancer [73], insulin-growth factor in lung cancer [74] p21 in ovarian cancer [75] and IKKα in nasopharyngeal carcinoma [76]. It should be noted though that EZH2 activity has also been associated with transcriptional activation of oncogenic signaling pathways associated with CSC survival and proliferation such as Wnt signaling in colorectal cancer [73, 77], NOTCH1 and TGFβ1 in prostate cancer [78], and STAT3 pathway in glioblastoma [65]. On the contrary, EZH2 inactivation correlates with increased growth and proliferation of stem-cells and early-progenitor-cells in early T cell precursor acute lymphoblastic leukemia (ETP-ALL), which represents an aggressive type of acute lymphoblastic leukemia [79]. It is intriguing to speculate that EZH2, as a chromatin modulator and part of PRC2, is involved in cancer stem cell maintenance through alteration of transcriptional programs.

### Cell cycle progression and EMT

One of the most important biologic roles of EZH2 in non-malignant cells is the regulation of cell cycle and it has been shown that EZH2 is required for the expression of genes implicated in E2F-driven cell proliferation. As mentioned above, E2F is involved in EZH2 deregulation in cancer and creates a double feedback between EZH2 and E2F-mediated cell proliferation and tumor progression. Moreover, down-regulation of EZH2 in glioblastoma leads to cell cycle arrest at the G0/G1 phase. This is associated with β-catenin/TCF4 and STAT3 downregulation, which can, in turn, suppress EZH2 expression representing a negative feedback loop that is deregulated in glioblastoma leading to uncontrolled cell cycle progression [80]. Thus, EZH2 significantly affects cell cycle progression in cancer cells.

It has been shown that PRC2 suppresses the Ink4a/Arf pathway which is known to mediate DNA damage repair signaling that is critical for the development and progression of several malignancies [81]. Similarly, it has been shown that EZH2 down-regulates the expression of RAD51, which is also critical for the DNA damage repair especially under hypoxic conditions in solid tumors [55]. These alterations lead to accumulation of genomic aberrations such as RAF1 gene amplification and activation of RAF1-ERK-β-catenin signaling promoting the aggressiveness of breast tumors [55]. These conclusions highlight the critical role of EZH2 as part of PRC2 on DNA damage repair, which is impaired in various malignancies leading to accumulation of DNA damage and genomic instability.

There is strong evidence that EZH2 promotes epithelial to mesenchymal transition (EMT) by interacting with SNAIL1 and down-regulating the expression of *E-cadherin* [82, 83]. EZH2 also mediates the silencing of the Disabled Homolog2-Interacting Protein (DAB2IP), which has been implicated in the regulation of EMT and metastatic potential in colorectal cancer [84] and is a biomarker of good prognosis in medulloblastoma [85]. According to these findings in multiple malignancies EZH2 mediates the activation of EMT program, which is a known mechanism inducing tumor aggressiveness and metastasis.

### Activation of oncogenic pathways

Recent studies have revealed that EZH2 upregulates oncogenic pathways acting independently of PRC2 at the transcriptional level. In prostate cancer cells, EZH2 interacts with androgen receptor (AR) independently of PRC2, and functions as a transcriptional co-activator inducing the expression of AR target genes [64]. Interestingly, in breast cancer EZH2 interacts with estrogen receptor (ER) and α and β-catenin promoting the expression of ER and Wnt signaling target genes [86]. It was also found that EZH2 interacts with RelA/RelB complex co-regulating a subset of NF-κB targets independently of PRC2, and increasing the aggressiveness of breast cancer cells [87]. EZH2 also activates Ras and NFκB signaling through down-regulating DAB2IP promoting prostate tumor development and metastasis [88]. These data suggest that EZH2 can act as a chromatin modulator independently of PRC2 inducing the expression of oncogenes involved in the development and progression of various cancers.

### Posttranslational modification of signaling molecules

Apart from its role as a chromatin modulator or direct activator of oncogenic pathways, EZH2 can alter signaling pathways in cancer cells by posttranslational mechanisms that alter the properties of key signaling molecules, further supporting the complexity of EZH2 involvement in cancer biology. For example, EZH2 can methylate non-histone substrates such as STAT3 inducing the tumorigenicity in glioblastoma stem-like cells [65] and melanoma [89]. EZH2 can also methylate AR in prostate cancer promoting its chromatin binding and the up-regulation of AR downstream targets [64]. Similarly, EZH2 directly methylates talin, a critical component of the cell migration machinery and disrupts talin binding to F-actin thereby disrupting the cell-to-cell adhesion process in leukocytes [90]. Although such talin-related function of EZH2 has not been identified in cancer cells, it would be important to investigate whether such mechanism mediated by EZH2 might affect cancer cell adhesion and migration and might be critical for the regulation of invasion and metastasis.

## INVOLVEMENT OF EZH2 IN TUMOR IMMUNITY

**Figure 3:**
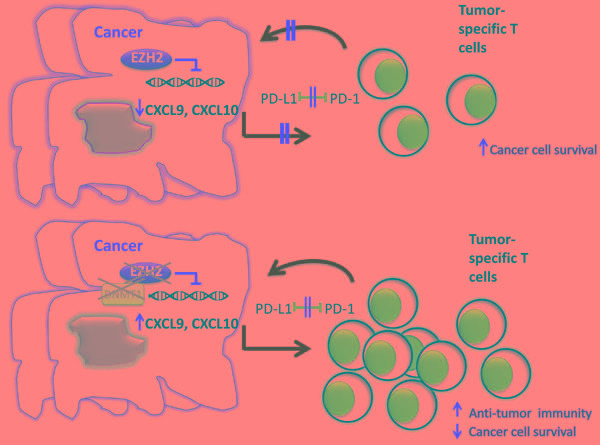
EZH2 expression and function in cancer cells alters the efficacy of tumor immunotherapy **A.** Expression and activation of EZH2 in cancer cells inhibits the production of Th1 type chemokines such as CXCL9 and CXCL10, thereby compromising the trafficking of tumor-specific T cells, which mediate cytotoxic effects on cancer cells and promote anti-tumor function. **B.** Inhibition of EZH2 together with DNMT1 promotes T cell recruitment to the tumor site and enhances responses to blockade of the PD1: PD-L1 checkpoint.

As mentioned above, EZH2 has been studied extensively as a promoter of cancer progression through the induction of cell cycle and inhibition of cancer cell differentiation. However, recent work revealed that EZH2 might have a novel and important involvement in cancer via a mechanism that alters the immunogenicity of the tumor microenvironment (Figure 3). In a recent study, Peng et al, using a mouse model of ovarian cancer, showed that epigenetic alterations of cancer cells by inhibition of EZH2 and DNA methyltransferase 1 (DNMT1) resulted in increased expression of the Th1-type chemokines CXCL9 and CXCL10 in cancer cells, leading to increased trafficking of effector T cells to the tumor site and decreased tumor volume [91]. Conversely, increased expression of EZH2 and DNMT1 in ovarian tumors was associated with decreased infiltration of CD8+ T cells and poor prognosis. Furthermore, treatment of ovarian tumors with EZH2 and DNMT1 inhibitors increased the efficacy of tumor-associated antigen-specific CD8+ T cells in response to inhibition of the PD-1: PD-L1 checkpoint [91]. A similar effect of EZH2 was observed in colorectal cancer, where targeting EZH2 in cancer cells also augmented the expression of CXCL9 and CXCL10 chemokines, affecting the infiltration of the tumor by effector T cells [92].

A recent study by Yin et al has provided insight to the contribution of epigenetic mechanisms in the development of NK cells and suggested that EZH2 inhibitors might inhibit tumor growth directly and indirectly through mobilization of NK cells. Ezh2-null hematopoietic stem and progenitor cells (HSPCs) or HSPCs treated with Ezh2 inhibitors gave rise to increased NK precursors and mature progeny that display enhanced cytotoxicity against tumor cells and these functional outcomes were associated with up-regulation of IL-15R (CD122) and the NKG2D-activating receptor [93]. Because EZH2 is overexpressed in several cancers, it has been considered that can function as a tumor-associated antigen (TAA). Indeed, identification of an immunogenic epitope of EZH2 recognized by CD4 T-cell in lung cancer, could serve as a potent immunogenic target inducing both CD4 and CD8 T-cell anti-tumor responses [94].

There is accumulating evidence that EZH2 has a direct role in T cell differentiation and function. EZH2 expression in naïve T cells inhibits Th1 and Th2 differentiation but is critical for survival, proliferation and efficacy of effector CD4+ and CD8+ T cells in vivo [95]. These observations suggest that while inhibition of EZH2 in cancer cells can promote anti-tumor immunity by enhancing recruitment of T effector cells in the tumor microenvironment [91, 92], inhibition of EZH2 on T cells may suppress survival, expansion and efficacy of such tumor-specific effectors thereby inducing the opposite effect. Indeed, Zhao et al determined that via competitive glucose consumption, ovarian cancer can induce expression of specific micro-RNAs in CD4+ and CD8+ T cells of the tumor microenvironment, which suppress the expression of EZH2 and decrease their survival and immune function [96]. Moreover, the authors demonstrated that down-regulation or inhibition of EZH2 in tumor-specific T cells increases the tumor burden and the metastatic potential in mouse models of ovarian cancer and melanoma, respectively. Importantly, the percentage of EZH2^+^ CD8^+^ T cells in the ovarian cancer tissues is a stronger predictor of overall and progression-free survival compared to the percentage of CD8^+^ T cells. These data support the hypothesis that EZH2 has a key role for the function of tumor-specific effector T cells, while cancer can evade tumor surveillance by targeting T-cell specific expression of EZH2 in the tumor microenvironment.

EZH2 also has an important role in the function of T regulatory cells (Tregs), which are also involved in immune homeostasis and anti-tumor immunity. It has been recently recognized that EZH2 is critical for the recruitment and homing of activated Tregs at the sites of inflammation [97]. Consistent with these findings, Yang et al determined that EZH2-deficient Tregs failed to protect mice from the development of autoimmunity in a model of naïve T cell-mediated colitis [95]. It has been documented that tumor cells can induce Tregs in the tumor microenvironment by secreting soluble factors such as TGF-beta, VEGF and GM-CSF, which convert tissue infiltrating CD4+ T cells to Foxp3+ Treg [98, 99]. Because of the unequivocal negative impact of Treg localization in the tumor microenvironment on anti-tumor immunity [100-103] the regulation of EZH2 expression in Tregs in tumor microenvironment is of particular interest for understanding and modulating anti-tumor immunity.

These results support the hypothesis that the tumorigenic effects of epigenetic modifications in cancer cells may be mediated by alteration of anti-tumor T cell function in the tumor microenvironment. Importantly, the functions of EZH2 in T effector and Treg cells appear to mediate distinct and opposing roles, in anti-tumor immunity. Thus, the use of EZH2 inhibitors with the goal to inhibit tumor growth will likely simultaneously alter the functions of immune cells in the tumor microenvironment. Although EZH2 inhibitors would provide an attractive treatment strategy in cancers with high EZH2 expression and activation, such approaches might have unpredictable effects on anti-tumor immunity.

## TARGETING OF EZH2 FOR CANCER THERAPY

Given the extensive implication of EZH2 on cancer development and progression, the introduction of EZH2 inhibitors has been an area of intense pre-clinical and clinical investigation. 3-deazaadenosine A (DZNep) is a non-specific EZH2 inhibitor which decreases its protein levels and removes the H3K27me3 marks from the chromatin and shows significant anti-tumor effect in various malignancies with reported efficacy but also toxicity in animal models [104, 105]. Numerous other agents such as ECGC, PL3 and CDF act similarly to DZNep decreasing the protein levels of EZH2 but due to their limited specificity, toxicity is the main concern and this is probably the reason why those agents have not been evaluated in clinical trials yet [106-108].

Recent studies have demonstrated that inhibition of the interaction between EZH2 and other components of the PRC2 complex may be more a specific approach with efficacy against various neoplastic diseases and decreased toxicity. Stabilized alpha-helix of EZH2 (SAH-EZH2) peptides disrupt the EZH2/EED interaction and decrease the EZH2 levels leading to decreased methyltransferase activity leading to growth arrest and differentiation of MLL-AF9 leukemic cells [109]. Similarly, Astemizole is a small molecule inhibitor of EZH2/EED interaction which destabilizes PRC2 complex decreasing the proliferation of lymphoma cells [110]. Finally, CPI-1205 selectively inhibits the interaction of EZH2 with PRC2 complex and is currently evaluated in a phase 1 clinical trial in patients with B-cell lymphomas (NCT02395601).

Currently, research is more focused on agents acting as competitive inhibitors of EZH2 binding to the wild type or the mutant protein. EPZ0005687 is a selective EZH2 inhibitor that can bind to the wild-type and the Y641 mutant EZH2. EPZ0005687 has shown efficacy in inhibiting the H3K27me3 activity in lymphoma, breast and prostate cancer cells [10, 38]. GSK126 is another EZH2 inhibitor, which appears to be effective against wild type and mutant EZH2 [111]. Particularly, it was demonstrated that GSK126 can effectively inhibit the proliferation of EZH2 mutant DLBCL both in vitro and in xenograft mouse models [39]. Similarly, it was found that inhibition of EZH2 with GSK126 induces apoptosis in multiple castrate resistant prostate cancer cell lines [112]. In addition, GSK126 can target mutant EZH2 effectively in melanoma cells by de-repressing tumor suppressor genes and subsequently inhibiting tumor growth [113]. Finally, it was recently reported that GSK126 can inhibit cell migration and angiogenesis in models of gastric and lung cancer [114] further supporting that this compound effectively suppresses the EZH2-mediated oncogenic pathways may be a promising agent in targeted therapeutics in cancer. Table 1 summarizes the currently available EZH2 inhibitors, their mechanism and the status of their clinical development.

**Table 1:** EZH2 inhibitors: Mechanism and status of clinical development

Compound	Mechanism	Status of development	References
DNZep	Regulator of EZH2 levels	Pre-clinical	[103, 104]
EGCG	Regulator of EZH2 levels	Pre-clinical	[105]
PL3	Regulator of EZH2 levels	Pre-clinical	[106]
CDF	Regulator of EZH2 levels	Pre-clinical	[107]
SAH-EZH2	Inhibitor of EZH2-PRC2 interaction	Pre-clinical	[108]
Astemizole	Inhibitor of EZH2-PRC2 interaction	Pre-clinical	[109]
CPI-1205	Inhibitor of EZH2-PRC2 interaction	Phase I (ClinicalTrials.gov Identifier NCT02395601)	
EPZ005687	Competitive EZH2 inhibitor	Pre-clinical	[10, 38]
GSK126	Competitive EZH2 inhibitor	Phase I clinical trial (ClinicalTrials.gov Identifier NCT02082977)	[39, 110-113]
EPZ011989	Competitive EZH2 inhibitor	Pre-clinical	[114]
ZLD1039	Competitive EZH2 inhibitor	Pre-clinical	[115]
EPZ-6438	Competitive EZH2 inhibitor	Phase II clinical trials (ClinicalTrials.gov Identifier NCT01897571)	[10, 116]

Last year, the development of EPZ011989, a selective and orally available EZH2 inhibitor, was reported and was shown to have significant activity in a mouse xenograft model of B cell lymphoma [115]. Another more recent study introduced ZLD1039, a highly selective, potent and orally available agent decreasing the H3K27 methylation leading to up-regulation of silenced tumor suppressor genes in breast cancer [116]. This compound can suppress the tumor growth but also inhibit metastasis in a mouse breast cancer xenograft model [116]. EPZ6438, another orally available EZH2 inhibitor, decreases the levels of H3K27me3 in a dose-dependent manner leading to significant reduction of tumor growth in non-Hodgkin lymphoma expressing EZH2 mutant [117]. EPZ6438 is currently under evaluation in a phase 1/2 clinical trial in patients with B cell lymphomas and advanced solid tumors (NCT01897571) with encouraging preliminary findings [10]. This compound is being evaluated in a phase 1 clinical trial for pediatric patients (NCT02601937) and in a phase 2 clinical trial for adults (NCT02601950) evaluating the efficacy and safety for patients with relapsed rhabdoid tumors, synovial sarcomas, renal medullary carcinoma and epithelioid sarcoma.

Numerous recent studies have concluded that EZH2 inhibition increases the sensitivity of cancer cells to radiation and traditional chemotherapy. Alimova et al have shown that EZH2 inhibition sensitizes atypical teratoid/rhabdoid tumor cells to radiation [118] while Xia et al demonstrated that down-regulation of EZH2 by siRNA enhances the anti-tumor effect of ionizing radiation in non-small cell lung cancer [119]. EZH2 inhibition in EGFR and BRG1 mutant lung cancer leads to accumulation of cancer cells in S phase, anaphase bridging and subsequently to increased sensitivity to Topoisomerase II inhibitors [120]. Inhibition of EZH2 in B cell lymphomas induces p53-mediated apoptosis under DNA damage accumulation, re-sensitizing these cells to etoposide [120, 121]. Combination of the EZH2 inhibitor GSK126 with etoposide in prostate cancer led to accumulation of DNA damage and increased the apoptotic rates compared to each monotherapy [122]. Recently, Neo et al identified c-Rel as a positive regulator of EZH2 expression in activated primary murine lymphocytes and human malignant lymphoid cells and treatment with the c-Rel inhibitor pentoxifylline (PTX) not only reduced EZH2 expression but also reduced the survival of human leukemia/lymphoma cell lines by enhancing their sensitivity to the EZH2-specific inhibitor, GSK126 [123]. These studies provide strong evidence that the addition of EZH2 inhibitors can increase the sensitivity of cancer cells to traditional therapeutic approaches, small molecule inhibitors or radiation therapy by increasing the levels of DNA damage and genomic instability, and further suggest the promising potential of EZH2-targeting compounds in in cancer therapeutics.

## EZH2 POLYMORPHISMS AND IMPLICATIONS IN CANCER BIOLOGY AND IMMUNOTHERAPY

Several EZH2 gene variations have been proposed to influence the risk of carcinogenesis, bearing either a predictive or a prognostic role. According to a recent study, the “CC” genotype of rs6950683 and rs3757441 is associated with decreased risk of oral squamous cell carcinoma development in Taiwan population, compared to carriers of the wild-type gene [124]. Additionally, EZH2 promoter hypermethylation has been observed in rs6950683 CC genotype in patients with oral squamous cell carcinoma (OSCC) [124]. Another study in Taiwan population suggested that the risk of urothelial carcinoma development is decreased by the presence of at least one C allele on the rs6950683 SNP, but the presence of at least one G is more common in urothelial carcinoma patients with less invasive tumor compared to patients without a G allele [125]. rs6950683 is also associated with liver carcinoma in Taiwan population, since the presence of at least a C at rs6950683 and rs3757441 decreases the risk of developing hepatocellular carcinoma but the same genotype simultaneously increases the risk of lymph-node-metastasis in hepatocellular carcinoma patients [126]. In Han Chinese population, 148505302C>T polymorphism seems to have a protective action against colorectal carcinoma, in contrast to 626-394T>C which presents an increased risk [127]. 626-394T>C is also associated with increased risk of esophageal squamous cell carcinoma in the same population [128]. Finally, reduced risk of gastric cancer is associated with rs2072407, rs734005, and rs734004, while rs12670401 and rs6464926 contribute to increased risk of gastric cancer occurrence [129].

According to Paolicchi et al, longer overall survival in cholangiocarcinoma patients is associated with rs887569 TT genotype compared to cholangiocarcinoma patients carrying CT or CC genotype [130]. In metastatic colorectal cancer patients treated with first-line 5-fluorouracil, folinic acid, irinotecan (FOLFIRI) and bevacizumab, shorter overall survival and progression-free survival was correlated with expression of rs3757441 C/C compared to rs3757441 T/T or C/T patients [131]. Moreover, EZH2 expression is higher in rs3757441 C/C genotype patients [131]. In addition, the risk of hepatocellular carcinoma is reduced in GC or GC rs2302427 genotypes carriers [132]. Finally, significantly reduced risk of lung cancer development is associated by rs6950683 and rs3757441 [133].

## CONCLUSIONS

EZH2 is a methyltransferase and catalytic component of the PRC2, and mediates methylation of H3K27. Accumulating evidence from in vitro and in vivo models together with studies in human cancers strongly suggest that EZH2 is involved in the development and progression of several human malignancies. EZH2 activity is up-regulated in cancer due to gain-of-function mutations, down-regulation of its suppressors, amplification of gene expression or increase of protein abundance by oncogenic pathways such as PI3K-AKT and MEK-ERK, post-transcriptional and post-translational modifications. EZH2 mediates its functions as part of PRC2 but also as an independent chromatin modulator. EZH2 downregulates the expression of tumor suppressor genes such as GSK-3β, RAD51 and P53 and upregulates oncogenes such as STAT3 and NOTCH1, promoting cancer cell survival and proliferation, increasing genomic instability and inhibiting cell differentiation. EZH2 has been particularly associated with the expansion of cancer stem cells, cell cycle progression, accumulation of DNA damage through inhibition of DNA damage repair and epithelial to mesenchymal transition. Targeted compounds inhibiting EZH2 have been introduced in cancer therapeutics with promising results in pre-clinical studies as monotherapies or in combination with traditional chemotherapy and are currently under evaluation in phase 1 and 2 clinical trials. EZH2 is critical for survival, proliferation and efficacy of effector CD4+ and CD8+ T cells but also promotes recruitment of Treg cells in sites of inflammation. Inhibition of EZH2 and DNA methyltransferase 1 (DNMT1) resulted in increased expression of the Th1-type chemokines in cancer cells, leading to increased trafficking of effector T cells to the tumor site and decreased tumor volume. As EZH2 inhibitors are currently being tested in several clinical trials as anti-cancer drugs, novel important observations are anticipated to emerge about how these compounds also affect anti-tumor immune function. In the era of cancer immunotherapy, therapeutic targeting EZH2 will pose new challenges.
